# Urinary Function following Laparoscopic Lymphadenectomy for Male Rectal Cancer

**DOI:** 10.1371/journal.pone.0078701

**Published:** 2013-11-12

**Authors:** Li-ye Liu, Wei-hui Liu, Yong-kuan Cao, Lin Zhang, Pei-hong Wang, Li-jun Tang

**Affiliations:** Department of General Surgery, Chengdu Military General Hospital, Chengdu, Sichuan Province, P.R. China; Virginia Commonwealth University School of Medicine, United States of America

## Abstract

**Objectives:**

Urinary function can be protected following open lateral node dissection (LND) with pelvic autonomic nerve preservation (PANP) for advanced rectal cancer. However data regarding urinary function after laparoscopic LND with PANP have not been reported. The goal of this study was to determine the effects of laparoscopic LND with PANP on urinary function in male patients with rectal cancer.

**Methods:**

Urine flowmetry was performed using an Urodyn flowmeter. Patients were also asked to complete the standardized International Prostate Symptom Score (IPSS) questionnaire before surgery and 6 months after. In total, this study consisted of 60 males with advanced rectal cancer.

**Results:**

No significant differences were seen in maximal urinary flow rate, voided volume or residual volume before and after surgery. The total IPSS score increased significantly after surgery and at least 41 patients (68.3%) reported there was no change in one of the seven IPSS questions.

**Conclusions:**

Laparoscopic LND with PANP was relatively safe in preserving urinary function.

## Introduction

It has been reported that the incidence of lateral lymph node metastasis ranges from 10 to 25% in patients with advanced middle and low rectal carcinomas [1]. It has also been suggested that lymphatic spread of cancer cells to lateral pelvic nodes may be a reason for local failure [2], [3]. Therefore, some authors have advocated that lateral nodes dissection (LND) is beneficial for local control and long-term survival. Many studies indicated that significant improvement in survival and a reduction in local recurrence can be achieved with LND [4], [5]. However, postoperative urinary dysfunction due to damage of the hypogastric nerves and pelvic nerve plexuses was observed in 42% to 73% of patients [6], [7]. The majority of surgeons have been reluctant to use LND due to the frequent complication of urinary dysfunctions. Recent studies suggest that the use of pelvic autonomic nerve preservation with lateral node dissection reduces the disturbance in male urinary function [8].

Over the past decade, advancements in surgical techniques and improved laparoscopic instruments have allowed most rectal cancer excision procedures to be performed laparoscopically. Compared to open surgery, laparoscopic rectal cancer resection is associated with less postoperative pain, faster return of bowel function, and shorter hospital stay [9], [10]. However, little is known about the incidence of urinary dysfunction after LND with PANP using laparoscopic technique. Theoretically, a magnified view of the pelvis may facilitate autonomic nerve identification. Consequently, lower levels of bladder dysfunction in men after laparoscopic procedures should be observed. Therefore, we conducted this study to test the male urinary function after laparoscopic LND with PANP for rectal cancer. We hypothesized that given the well-illuminated magnified view of laparoscopy, the autonomic nerves and male urinary function can be preserved.

## Materials and Methods

### 1. Patients

This study was performed between October 2010 and October 2012. The present study conformed to the ethical standards of the World Medical Association Declaration of Helsinki and we get the permission of Chengdu Military General Hospital Medical Ethics Committee (Register Number: 2010051). All patients had signed informed consent form. All patients underwent laparoscopic LND with PANP. Exclusive criteria were as follows: (1) those with intestinal obstruction requiring urgent decompression, (2) males with stage 0, stage I and stage IV tumors with lung metastasis assessed by transrectal ultrasonography upper abdomen and pelvic enhancement CT scan, (3) those with a contraindication to general anesthesia under pneumoperitoneum, (4) those who were obese (body mass index≥30 kg/m^2^), (5) tumors located in the upper third of the rectum. The rectum was divided into three parts: the lower third (within 7 cm of the anal verge), the middle third (8–12 cm), and the upper third (13–16 cm). This study included 60 rectal cancer patients who were diagnosed with cancer in the mid or lower rectum. All patients underwent preoperative tumor staging with a contrast medium enema, rectoscopy and colonoscopy with biopsies of the tumor, endorectal ultrasonography, abdominal ultrasound, and a chest x-ray.

Patients with locally advanced rectal carcinoma (uT3/uT4) and no evidence of distant metastases were candidates for neoadjuvant chemoradiation with the following schedule: 30–50 Gy radio-therapy and 5-fluouracil (5-FU) combined with folinic acid over 5 weeks. The operation was carried out two to three weeks after completion of the multimodality treatment. Adjuvant treatment was administered to patients with UICC stage III disease and consisted of six cycles of 5-FU. Characteristics of these patients are listed in Table 1.

**Table 1:** Patient characteristics.

Characteristics	No. of Patients (n = 60)
Age, yr (mean ± standard deviation)	54.6±10.3
Type of operation(no)	
Mile’s procedure	17
Dixon’s procedure	43
Tumor size, cm^2^ (mean ± standard deviation)	12.23±8.7
Tumor location(no)	
Middle(8–12 cm)	34
Low(<8 cm)	26
TNM tumor stage(no)	
Stage III	43
Stage IV	17
Adjuvant treatment(no)	
Postoperative chemoradiotherapy	38
No adjuvant therapy	12

### 2. Assessment of Urinary Function

Urinary function was determined based on the following parameters: catheter indwelling, urine flow rate (Urodyn®, Dantec, Copenhagen, Denmark), recatheterization rate, long-term catheterization rate (beyond the day of discharge), and International Prostate Symptom Score (IPSS) [11]. The IPSS is subdivided into seven items: incomplete bladder emptying, frequency, intermittency, urgency, weak stream, straining and nocturia. The scoring system is based on a scale from zero to five as follows: 0, not at all; 1, less than one time in five; 2, less than half the time; 3, about half the time; 4, more than half the time; and, 5, almost always. In addition, another question, quality of life due to urinary symptoms, was also included in this questionnaire. The total score is calculated by adding the individual scores of each subdivision. Deterioration of postoperative urinary function was categorized into three groups: mildly symptomatic (IPSS 0–7 points), moderately symptomatic (IPSS 8–19), and severely symptomatic (IPSS 20–35 points). We defined it “no change” if the patient’s total IPSS score at the same category after and before surgery. The IPSS questionnaires were distributed and collected by the author at the outpatient clinic. Urinary flowmetry and IPSS were performed before surgery and 6 months after surgery.

### 3. Surgical Treatment

**Figure 1:**
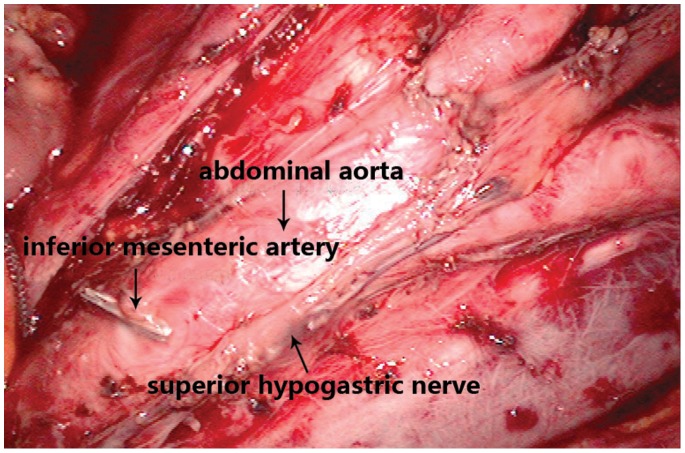
Preservation of the superior hypogastric nerve when removing lymph nodes. It is important to note that, superior hypogastric nerve is likely to be treated as connective tissue and be cut off in this procedure. Advantages of laparoscopy play an important role in this situation. Amplification of laparoscopy makes it easy to identify superior hypogastric nerve and thus damage could be avoided.

Autonomic nerve preservation consists of the identification and preservation of the superior hypogastric (sympathetic) and sacral splanchnic (parasympathetic) nerve trunks (Fig. 1), in addition to the pelvic autonomic nerve plexus. The plane of pelvic dissection was along the parietal pelvic fascia, leaving the hypogastric nerve trunk over the aorta. The left and right hypogastric nerves were identified at the level of aortic bifurcation. The lateral ligament was divided, separating the intact mesorectum from the pelvic autonomic nerve plexus. These procedures leave the sacral splanchnic nerve and the pelvic autonomic nerve plexus undamaged on the lateral pelvic wall. At the level of the fourth sacral vertebra, the rectosacral fascia was sharply divided to creating an opening in the retrorectal space up to the anal sphincter. In all cases, a clear oncologic distal and lateral margin was obtained after completion of pelvic dissection.

**Figure 2:**
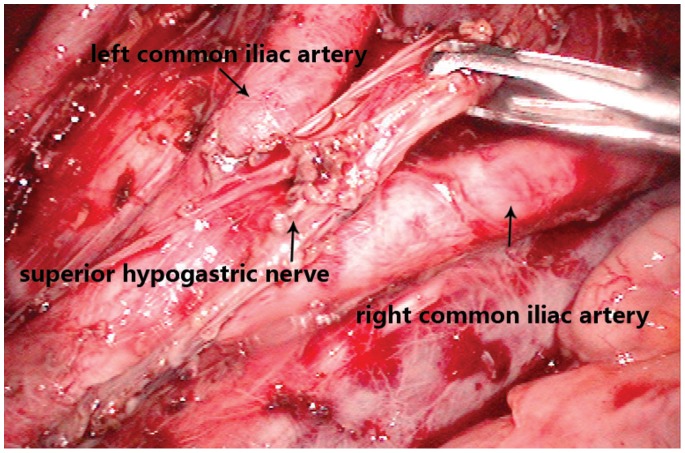
Preseration of autonomic plexus after removal of lymph nodes. Lymph nodes around the common, internal, and external iliac arteries should be removed in this procedure. Ureter should be identified clearly. Autonomic plexus walking in the middle of both sides of the common iliac artery, this feature should be noted during lymp nodes dissection.

**Figure 3:**
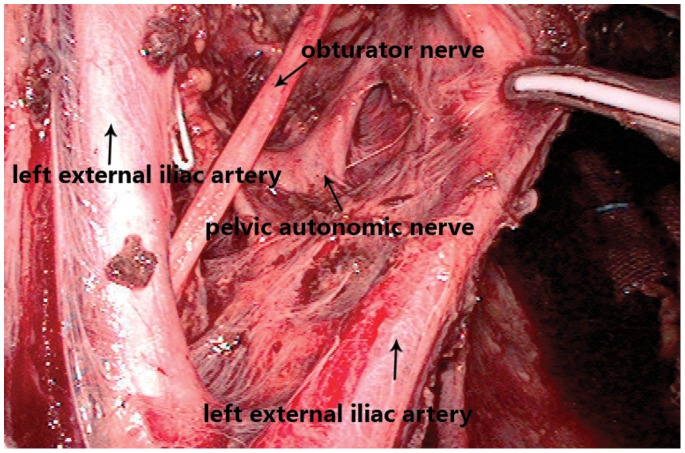
Preservation of pelvic autonomic nerve plexus when dissecting the obturator lymph nodes. This procedure is difficult not only because of the narrow space, but also concentrated pelvic autonomic nerve. Operation by laparoscopic technique here can occupy small space and identify tissues clearly. By this technique, the purpose of lymph nodes remove and autonomic nerve preservation could be achieved.

LND was performed by dissecting the lymph nodes around the common, internal, and external iliac arteries (Fig. 2), the middle rectal artery root, and the obturator space (Fig. 3). After the tumor was localized in the right or left side, ipsilateral LND was performed (n = 44). If the tumor was positioned anteriorly, posteriorly, or circumferentially, both sides of lateral lymph nodes were dissected (n = 16). Complete PANP was performed in 42 of 60 LND patients. Remaining patients had unilateral PANP. Average number of lymph nodes removed was 23(19 to 26).

### 4. Statistical Analysis

Statistical analysis was performed with SPSS 11.0(Chicago, IL). Nominal data were analyzed by test or Fisher’s exact probability. Differences in continuous variables were analyzed by Student’s *t*-test. Analysis of variance and multivariate analysis was also performed. P values were considered statistically significant when 0.05 or less.

## Results

### 1. Removal of the Catheter and Successful Voiding

On average, the indwelling catheter was removed 5.6 days after each operation. Most patients voided urine spontaneously. Three patients required recatheterization, but satisfactory voiding was noted within seven days of surgery. No patient was discharged with serious complications related to voiding function.

### 2. Urine Flowmetric Data

Three factors must be considered when assessing urine flowmetric data: maximal flow rate (Qmax, ml/s), voided volume (Vcomp, ml), and residual volume (RV, ml). Based on urine flowmetry, no significant differences were seen in maximal urinary flow rate, voided volume, or residual volume before and after surgery (20.9±4.3 vs. 19.8±3.9, 222.8±67.1 vs. 211.5±77.2, 3.1±1.6 vs. 4.6±1.5, mean ± standard deviation, *P*>0.05, respectively; Table 2). Tumor size, tumor location, and adjuvant chemoradiotherapy did not significantly affect urine flowmetric findings after surgery.

**Table 2:** Urine flowmetric findings before and after surgery (n = 60).

Items	Before surgery	After surgery	*P*
Qmax(ml/s)	20.9±4.3	19.8±3.9	0.12
Vcomp(ml)	222.8±67.1	211.5±77.2	0.39
RUV(ml)	3.1±1.6	4.6±1.5	0.17

Figures are mean ± standard deviation.

Qmax, maximal flow rate.

Vcomp, voided volume.

RV, residual volume.

### 3. IPSS

Total IPSS score (N = 60) was 6.6±4.7 (preoperatively) vs. 8.5±5.9 (postoperatively; mean ±standard deviation, *P*<0.05). The quality of life due to urinary symptoms score increased from 1.7±1.1 to 2.8±1.6 (mean ±standard deviation, *P*<0.05; Table 3) in patients preoperatively and postoperatively, respectively. The scores for each of the seven subsets of the IPSS (incomplete emptying, frequency, intermittency, urgency, weak stream, straining, and nocturia) did not show any significant differences after surgery. Further statistical analysis of the factors affecting the the IPSS showed age greater than 60 years (incomplete emptying, *P* = 0.021; frequency, *P* = 0.014) and tumor diameter of 4.5 cm or larger (weak stream, *P* = 0.033) were the only significant factors affecting the IPSS. Results of the IPSS questionnaire showed that there were no changes in incomplete emptying for 47 (78.3%) patients, in frequency for 42 (70.0%) patients, in intermittency for 51 (81.0%) patients, in nocturia for 44 (73.3%) patients, in urgency for 41 (68.3%) patients, in weak stream for 45 (75.0%) patients, and in straining for 55 (91.7%). (Table 4).

**Table 3:** Changes in IPSS before and after surgery.

Items	Before operation(n = 60)	After operation(n = 60)
Incomplete emptying	0.7±1.2	1.3±1.5
Frequency	0.8±1.1	1.2±1.2
Intermittency	0.8±0.9	1.4±1.5
Urgency	0.8±1.1	1.6±1.4
Weak stream	1.1±1.4	1.8±1.4
Straining	1.1±1	1.0±1.3
Nocturia	0.9±0.9	1.5±1.2
Total IPSS score	6.6±4.7	8.5±5.9^*^
Quality of life due to urinary symptoms	1.7±1.1	2.8±1.6^*^

Figures are mean ± standard deviation, *P = .001.

IPSS, International Prostate Symptom Score.

**Table 4:** Changes in postoperative urinary function measured by the IPSS.

Items	Absence (n = 60)
Incomplete emptying	47 (78.3)
Frequency	42 (70.0)
Intermittency	51 (81.0)
Urgency	41 (68.3)
Weak stream	45 (75.0)
Straining	55 (91.7)
Nocturia	44 (73.3)

Figures are numbers and (percentage).

IPSS, International Prostate Symptom Score.

## Discussion

Intrapelvic lymph node dissection for rectal carcinoma was first reported in the early 1950 s [12]. However, this technique was abandoned for a long period of time until late 1980 s. This was likely due to its technical difficulty, higher morbidity, and lack of evidence suggesting improved oncological outcomes. In the western countries, TME (total mesorectal excision, TME) has been widely accepted as the gold standard to reduce local recurrence [13], [14]. However, in locally advanced middle or low rectal carcinomas, approximately 10–25% of the tumors had lateral or skip lymph node metastasis [1], [15]. Although most of Western surgeons suggest that locally advanced rectal carcinoma is a systemic disease [16], [17], it is suggested that lateral node dissection may be beneficial for certain subgroups of patients [4], [18], [19].

Avulsion or direct injury to the hypogastric nerve plexus and the sacral nerve plexus during blunt pelvic dissection of the rectum is closely related to postoperative urinary dysfunction [20]. Hypogastric nerve injury results in the failure of complete bladder filling in men. Injury to the sacral parasympathetic nerves results in a loss of the urge to urinate. Adding complicated factors such as obesity or a narrow pelvis makes intraoperative nerve identification more difficult. This can potentially lead to nerve injury and, consequently, functional complications. Therefore, preservation of the pelvic autonomic nerves represents a key element in the preservation of urinary function [21], [22].

Our experience showed that laparoscopic LND and PANP can be performed safely and effectively by surgeons who have adequate experience with laparoscopic technique. Laparoscopic LND and PANP have several advantages. The laparoscopic magnification of the operative field helps the operator to identify the plane of loose connective tissue between the visceral and the parietal pelvic fascia [23]. A 30° laparoscope can reach the narrow lesser pelvis, resulting in improved vision and contributing a magnifying effect that makes it easier for the surgeon to identify and protect the pelvic autonomic nerve fibers and plexus.

It is extremely important for surgical treatment of rectal cancer to achieve a favorable oncologic outcome and to preserve function. Laparoscopic LND and PANP are based on clear anatomic knowledge of the pelvis, the ability to carefully remove lymph nodes, and skillful use of laparoscopic technique. In addition, pelvic autonomic nerve preservation must be performed completely. Therefore, the ultimate goals of these techniques are to prevent local failure and to preserve urinary and sexual function. Urinary dysfunction is a well-recognized complication of rectal cancer surgery. Urinary complications after conventional surgery have been observed in 42–73% of patients, and neurogenic bladder dysfunction was encountered in 16% of patients [24]. In the past 30 years, great strides have been made in understanding the importance of autonomic nerve preservation. This has led to a reduction in urinary dysfunction. Procard provided urodynamic profiles that demonstrated no change in urinary function following nerve-sparing TME [25]. Kyo and colleagues [26] reported that after LND and PANP, no severe dysfunction, such as major incontinence or catheterization, was observed. In our study, there were no significant complications related to voiding function at the time of discharge. There were also no significant differences in maximal flow rate, voided volume, and residual volume before and after surgery. But all of the three factors were slightly worse after surgery, indicated that although PANP technique was employed, urinary function may still changed after surgery in some cases. The possibly reason may be that amplification of the role of laparoscopy is limited, some of the small nerve fibers may be damage. Another reason was backward tilt after surgery may cause bladder neck obstruction and thus leading to bladder dysfunction [27]. In our series of patients, even though total IPSS was increased postoperatively, more than 70 percent of patients reported that they had no change in voiding function based on the seven items of the IPSS questionnaire. And we noticed that, quality of life due to urinary symptoms was worse after surgery than before. Patients with varying degrees of changes in symptoms of urinary function tended to complaint their quality of life due to urinary symptoms declined after surgery. Liang [28] reported that the voiding function after laparoscopic TME were good in 71.6% patients, fair in 23.0%, and poor in 5.4%. Kneist [22] found a significant difference in IPSS score after TME with PANP by open technique. Another study [29] shows that there was no significant difference in total mean IPSS score after laparoscopic TME, and quality of life due to urinary symptoms significantly decrease, but all other IPSS scores (incomplete bladder emptying, intermittency, urgency, weak stream, straining and nocturia) did not show significant changes at 15 months follow-up. Based on our data, laparoscopic LND with PANP was relatively safe in preserving urinary function. In addition, factors influencing the IPSS items were age greater than 60 years, and tumor size greater than 4.5 cm in diameter. Type of operation, TNM stage and adjuvant treatment had no impact on IPSS score.

Our data suggest that laparoscopic lateral node dissection with pelvic autonomic nerve preservation may provide acceptable nerve preservation according to urine flowmetric findings, eventhough the total IPSS score was higher after surgery. In addition, surgically acquired bladder dysfunction was affected by clinically pertinent factors such as age and tumor size, but it was not enhanced by laparoscopic surgery. The proposed advantages of laparoscopic technique, including the improved visibility afforded by a magnified, well-illuminated field of view, appear to aid the surgeon when confronted with technical surgical difficulties.
